# Time to match; when do homologous chromosomes become closer?

**DOI:** 10.1007/s00412-022-00777-0

**Published:** 2022-08-12

**Authors:** M. Solé, J. Blanco, D. Gil, O. Valero, B. Cárdenas, G. Fonseka, E. Anton, Á. Pascual, R. Frodsham, F. Vidal, Z. Sarrate

**Affiliations:** 1 Genetics of Male Fertility Group, Unitat de Biologia Cel·lular, Universitat Autònoma de Barcelona, Cerdanyola del Vallès, Spain; 2 Computer Vision Center, Computer Science Department, Universitat Autònoma de Barcelona, Cerdanyola del Vallès, Spain; 3 Servei d’Estadística Aplicada, Universitat Autònoma de Barcelona, Cerdanyola del Vallès, Spain; 4 Cytocell Ltd, Cambridge Science Park, Milton Road, Cambridge, CB4 0PZ UK

**Keywords:** Homologous chromosomes, Homologous pairing, Chromosome territories, FISH, Meiosis, Premeiotic cells

## Abstract

In most eukaryotes, pairing of homologous chromosomes is an essential feature of meiosis that ensures homologous recombination and segregation. However, when the pairing process begins, it is still under investigation. Contrasting data exists in *Mus musculus*, since both leptotene DSB-dependent and preleptotene DSB-independent mechanisms have been described. To unravel this contention, we examined homologous pairing in pre-meiotic and meiotic *Mus musculus* cells using a three-dimensional fluorescence in situ hybridization-based protocol, which enables the analysis of the entire karyotype using DNA painting probes. Our data establishes in an unambiguously manner that 73.83% of homologous chromosomes are already paired at premeiotic stages (spermatogonia-early preleptotene spermatocytes). The percentage of paired homologous chromosomes increases to 84.60% at mid-preleptotene-zygotene stage, reaching 100% at pachytene stage. Importantly, our results demonstrate a high percentage of homologous pairing observed before the onset of meiosis; this pairing does not occur randomly, as the percentage was higher than that observed in somatic cells (19.47%) and between nonhomologous chromosomes (41.1%). Finally, we have also observed that premeiotic homologous pairing is asynchronous and independent of the chromosome size, GC content, or presence of NOR regions.

**Supplementary Information:**

The online version contains supplementary material available at 10.1007/s00412-022-00777-0.

## Introduction

In meiosis, germ cells are subjected to profound chromosomal and morphological changes during the production of highly differentiated haploid cells. A key step in this process is the segregation of homologous chromosomes at anaphase I. In most eukaryotes, homologous segregation depends on the formation of bivalents in prophase I and the orientation of homologous centromeres towards opposite poles in the meiosis I spindle.

Bivalent formation begins at the early stages of meiosis, where each chromosome approaches and aligns with its homolog in order to carry out pairing, synapsis, and recombination (for a review, see Zickler and Kleckner 2015). Although pairing and synapsis are somehow related processes, specific differences must be mentioned when referring to meiotic analysis; the approaching, juxtaposition, and overlapping of homologous chromosomes are commonly referred to as *pairing*, meanwhile, the closer alignment and connection of homologous chromosomes by the synaptonemal complex is generally called *synapsis* (Zickler and Kleckner 2015).

The processes of synapsis and recombination have been extensively studied and described elsewhere (Zickler and Kleckner 1998, 1999; Champion and Hawley 2002; Page and Hawley 2003, 2004). In contrast, the mechanisms underlying the regulation and timing of the pairing process remain poorly understood, with studies demonstrating conflicting results and leaving many open questions.

Two pieces of data suggest that pairing begins during the leptotene stage of prophase I. First, there is a certain consensus that in many organisms, repair of the double-strand breaks (DSBs) produced by the topoisomerase-like protein SPO11 during leptotene is the inducing factor for homologous pairing in meiosis (reviewed by Baudat et al. 2013). Using various models, researchers have described associations between the levels of or the correct development of DSBs, and the extent of homologous coalignment, successful pairing, and/or synapsis (Thorne and Byers 1993; Romanienko and Camerini-Otero 2000; Davis et al. 2001; Grelon et al. 2001; Peoples et al. 2002; Tessé et al. 2003; Henderson and Keeney 2004; Kauppi et al. 2013; Rockmill et al. 2013). Secondly, during leptotene, the protein SUN1 tethers the chromosome ends to the inner nuclear envelope, where they cluster to form a structure that resembles a bouquet (Ding et al. 2007; Hiraoka and Dernburg 2009). Bouquet formation have been described in many species (Zickler and Kleckner 1998, 2015; Scherthan 2001; Harper et al. 2004), suggesting that they ease homology searching by bringing the ends of chromosomes closer and aligning them. In fact, lack of SUN1 causes asynapsis and gametogenesis disruption in mice (Ding et al. 2007; Chi et al. 2009). Accordingly, it has been postulated that, in the nuclei of premeiotic cells (before DSB and bouquet structure formation), homologous chromosomes are spatially separated from each other, as they are in somatic cells. This interpretation is experimentally supported by studies analyzing the chromosome distribution of homologs in premeiotic cells using fluorescence in situ hybridisation (FISH)-based strategies (Scherthan et al. 1996; Scherthan and Schönborn 2001).

Data from other studies suggests the existence of a mechanism of homolog pairing that takes place before DSB formation. The DSB-independent pairing mechanism has been observed in species with specific chromosomal characteristics, such as dipterans, which exhibit somatic chromosome pairing (Wandall and Svendsen 1985), and wheat, which has a hexaploid karyotype that requires specific genes to ensure meiotic pairing (Martinez-Perez et al. 2001; Martínez-Pérez et al. 1999; Prieto et al. 2004). However, this mechanism has also been observed in species without any distinctive chromosomal features, such as worms (Dernburg et al. 1998; Phillips et al. 2009), flies (McKim et al. 1998), budding yeast (Burgess et al. 1999; Cha et al. 2000), fission yeast (Scherthan et al. 1994; Nabeshima et al. 2001), and mice (Boateng et al. 2013). Although no clear molecular explanation has been established for this DSB-independent homolog recognition, research has suggested that heterochromatin aggregation, the SPO11 and SUN1 proteins, as well as some noncoding RNA and transcription factors, may be involved in the process (Page and Hawley 2004; Barzel and Kupiec 2008; Dombecki et al. 2011; Ding et al. 2012; Boateng et al. 2013).

Therefore, we cannot discard the coexistence of both DSB-dependent and -independent models of pairing according to species, and it is important to highlight that both models have been reported to occur in mice. This contrasting situation brought us to revaluate the timing of homologous meiotic pairing in mice germ cells via an experimental design based on the use of painting probes and a three-dimensional (3D) FISH strategy to analyze each chromosome.

## Methods

### Germ cell analysis

The methodology used to process germ cells has been previously described by our research group (Solé et al. 2021) and it consists of several steps:

- ***Cell obtainment***

Testicular tissue was obtained from four 12-week-old C57BL/6 J mice. The tissue was mechanically and enzymatically disaggregated following the procedure described by Garcia-Quevedo et al. (2012). Testicular cells were adhered to customized polylysine-coated slides (1 mg/ml), fixed with 4% paraformaldehyde, and subjected to a permeation treatment with 0.1 N hydrochloric acid, 0.5% triton, liquid nitrogen, and 0.005% pepsin (protocol adapted from Cremer et al. 2008). Prior to application of the FISH procedure, slides were incubated in 50% formamide for a minimal period of 2 months, with the purpose of achieving a prehybridization slow DNA denaturation.

- ***Fluorescence *in situ* hybridization***

Three successive rounds of FISH were performed using a custom designed Chromoprobe Multiprobe® OctoChrome Murine System ™ kit (Cytocell Ltd, Cambridge, UK). This kit contains a multiprobe device that consists of three different coverslips with seven delimited independent regions. Each of these regions presents a specific combination of three painting labeled with a different fluorochrome (Aqua DEAC, FITC, and Texas Red). Therefore, after sequential application of three coverslips, it is possible to identify up to nine chromosomes per region in the same nuclei. Analysis of all the coverslips and regions provides identification of all chromosomes of the mouse karyotype as well as all possible combinations of chromosome pairs.

In each round of FISH, the slide and the corresponding coverslip were mounted together in formamide solution and then subjected to denaturation for 5 min at 75 °C in a Hybridization Vysis HYBrite System. Hybridization was performed for 60 h at 37 °C (procedure adapted from Cytocell manufacturer instructions). After hybridization, the coverslip was removed and slides were transferred to 1 × saline-sodium citrate (SSC) buffer, incubated for 2 min, and washed in 2 × SSC/0.05% Tween-20 at RT for 30 s. Finally, hybridized areas were mounted in the antifade provided by the kit. After each FISH round, a washing step was performed with 0.0625 × SSC at 73 °C for 5 min in order to remove previous hybridization signals.

- ***Image capture***

**Fig. 1:**
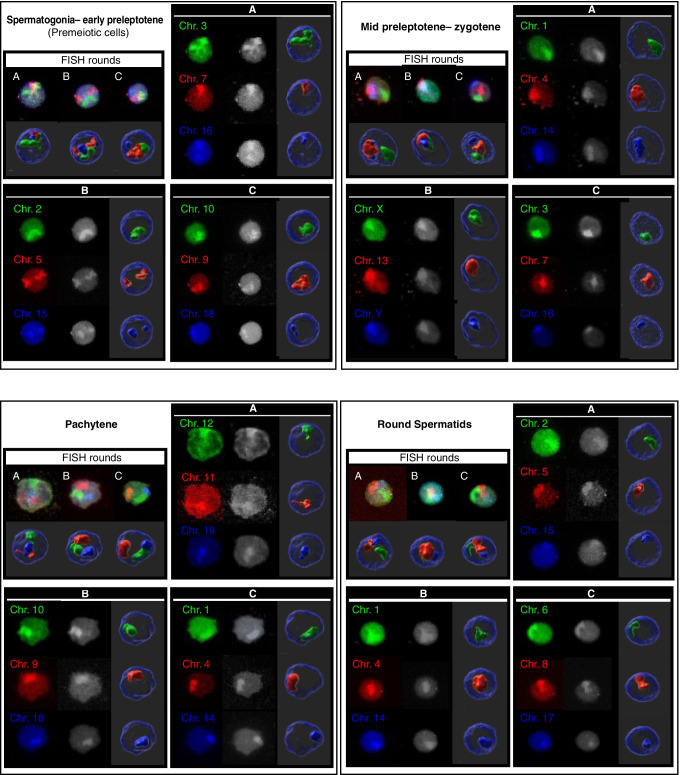
3D-FISH confocal image captures of each round of hybridization (**a**, **b**, and **c**) for four different mice germ cell nuclei (spermatogonia-early preleptotene, mid-preleptotene-zygotene, pachytene, and round spermatids). Different combinations of three chromosomes (Chr.) displayed in FITC, Texas Red, or Aqua DEAC are observed in each nucleus. For each hybridization round are shown maximum intensity projections of confocal serial sections and 3D composite reconstructions using Imaris 9.3 software in both RGB merge and split channels (to view the planes’ sequence of the maximum intensity confocal images and the 3D composite reconstruction of premeiotic cells, see Online resource 1)

A Leica TCS-SP5 confocal microscope coupled to an image analysis system (LAS AF v.1.8.1) was used to capture serial optical sections of nuclei after each hybridization round (Fig. 1). A hybrid detector (HyD) and HCX PL APO lambda blue 63.0 × 1.40 OIL UV objectives were used. Specifically, lasers and excitation frequencies applied were a 405 nm UV diode laser, a 488 nm argon laser, and a 561 nm DPSS laser for the Aqua DEAC, FITC, and Texas Red fluorochromes, respectively. The HyD detector was configured at 415–470 nm for Aqua DEAC, at 500–550 nm for FITC, and at 571–750 nm for Texas Red. A high-speed resonant scanner module was used to obtain serial optical sections on the *X*, *Y*, and *Z* axes, with a distance between sections of 0.17 μm, a 512 × 256 pixel format, and an optical zoom of 5X. The number of sections was different for each nucleus (i.e., according to the corresponding nuclear volume). All captures were associated with their coordinates in order to relocate and capture the same nuclei after each hybridization round.

- ***Image analysis***

Image analysis was performed using customized developed scripts designed within the Fiji software environment (Schindelin et al. 2012) and Matlab R2013b. The Fiji scripts permitted segmenting nuclei and chromosome territories in different serial binary images as shown in the animation Online resource 1. Following this, Matlab scripts permitted 3D reconstruction of the nuclei and chromosome territories, enabling the extraction of numerical data concerning chromosome position and volume, as well as nuclei volume.

We considered that two homologous chromosomes were paired when all pixels of a specific signal formed a unique and continuous group of voxels (Online resource 2). Conversely, homologous chromosomes were classified as unpaired when two groups of voxels were observed as two separate entities, so sharing no voxel (Online resource 2). Similarly, we considered that two nonhomologous chromosomes (also referred to as heterologous chromosomes) were associated when their territories overlapped. The percentage of overlapping was determined as the number of shared voxels respect the voxels each chromosome territory occupy.

- ***Identification of cell type***

**Fig. 2:**
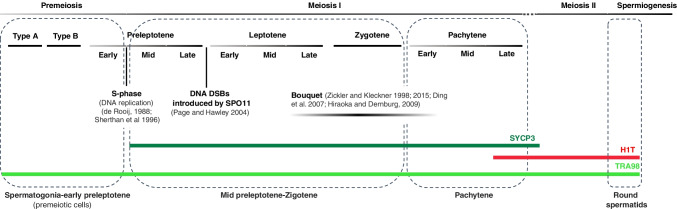
Graphical representation of the labeling pattern of synaptonemal complex protein 3 (SYCP3), testicle-specific histone 1 (H1T), and a testis-specific nuclear protein known as TRA98 during the spermatogenesis process, along with some relevant meiotic events that occur in primary spermatocytes. The colored bars indicate the presence of each protein: SYCP3 (dark green bar), H1T (red bar), and TRA98 (light green bar), throughout spermatogenesis (the top grey bar)

An immunofluorescence procedure was designed to unequivocally identify premeiotic cells in relation to somatic cells and meiotic cells. With this purpose, three proteins were identified: synaptonemal complex protein 3 (SYCP3), which allowed identification of germ cells from mid-preleptotene to pachytene stages; testicle-specific histone H1 (H1T), which enabled identification of germ cells ranging from the late pachytene stage to round spermatids; and a germ cell-specific nuclear antigen recognized by the monoclonal antibody TRA98 (Tanaka et al. 1997), which allowed discrimination between somatic cells and germ cells. Various cell fractions were then identified based on the presence or absence of these proteins as previously shown by our research group (Solé et al. 2021):

- I. Premeiotic cells. This category includes cells from A-type spermatogonia to early preleptotene spermatocytes (the onset of the premeiotic S-phase in which DNA replication is performed). These nuclei stained positively for TRA98, with negative staining for SYCP3 and histone H1T (Fig. 2).
- II. Mid preleptotene-zygotene spermatocytes. These nuclei showed positive staining for TRA98 and for SYCP3 protein and negative staining for histone H1T (Fig. 2). Despite using the same fluorophores to detect SYCP3 and TRA98, both proteins were easily distinguishable because TRA98 showed a uniform labeling pattern that was distinct from SYCP3’s dotted or thread-like appearance (Fig. 2).
- III. Pachytene spermatocytes. Pachytene spermatocyte nuclei stained positive for TRA98, demonstrated a thread-like staining pattern for SYCP3, and were either negative (early pachytene) or positive (late pachytene) for histone H1T (Fig. 2).
- IV. Round spermatids. Spermatid nuclei stained positive for TRA98 and histone H1T and were negative for SYCP3 (Fig. 2).

The protein labeling process was carried out after chromosomal FISH analysis in two sequential rounds. In the first round, detection of SYCP3 and histone H1T was performed while, in the second round, samples were immunostained with the monoclonal antibody against TRA98. Briefly, cells were fixed in 3% paraformaldehyde for 10 min and permeabilized in a 1 × PBS-0.5% Triton-X100 solution for 5 min. Next, samples were incubated for 15 min with blocking solution (1% w/v bovine serum albumin). After blocking, the cells were incubated at 4 °C with the primary antibodies, specifically rabbit anti-SCP3 (Abcam, Cambridge, UK) plus either guinea pig anti-H1T (The Jackson Laboratory, USA) (first round) or rat anti-TRA98 (Abcam, Cambridge, UK) (second round). Next, samples were incubated with secondary antibodies for 40 min at 37 °C. Specifically, the secondary antibodies were goat anti-rabbit FITC (Jackson ImmunoResearch Inc., Cambridge, UK) and goat anti-guinea pig CY3 (Jackson ImmunoResearch Inc., Cambridge, UK) (first round) or goat anti-rat FITC (Abcam, Cambridge, UK) (second round).

- ***Data processing and statistical analysis***

Results were analyzed and processed in the following manner: data regarding chromosome position and volume, which were extracted from the application of Matlab scripts, permitted to calculate (1) the average percentage of paired and unpaired homologous chromosomes; (2) the average percentage of nonhomologous chromosomes sharing the same chromosomal territory; (3) the average of nonhomologous overlapping percentage; and (4) the nuclear volume proportion occupied for each chromosomal territory analyzed.

Pearson’s correlations were performed to evaluate the degree of linear relationship between chromosome size, GC content, and chromosome gene density (these parameters are detailed on Table 1) and the average rate of homologous chromosome pairing. Besides, the percentage of paired homologous chromosomes was compared between NOR-bearing-chromosomes and no NOR-bearing-chromosomes by a *T*-test. These analyses were only performed at the first two stages studied (i.e., spermatogonia-early preleptotene spermatocytes and mid preleptotene-zygotene spermatocytes), as there were no unpaired chromosomes in the remaining stages.

**Table 1:** Chromosome features used to test for possible conditioning factors of homologous pairing. Data extracted from The Genome Reference Consortium, *Mus musculus* GRCm38.p6. ^a^Data extracted from The Genome Reference Consortium, *Mus musculus* GRCm38.p6. ^b^Data extracted from (Evans et al. 1974; Henderson et al. 1974; Atwood et al. 1976; Dev et al. 1977; Kurihara et al. 1994; Britton-Davidian et al. 2011). Crosses (x) indicate the presence of nucleolus organizer regions (NORs)

Chr	Size (Mb)^a^	% GC^a^	Gene density^a^	NOR^b^
1	195.47	41.3	2,687	
2	182.11	42.2	3,491	
3	160.04	40.7	2,225	
4	156.51	42.5	2,622	
5	151.84	42.7	2,507	
6	149.74	41.6	2,597	
7	145.44	43.2	3,798	
8	129.40	42.6	2,177	
9	124.60	42.9	2,276	
10	130.70	41.6	2,086	
11	122.08	44.0	2,852	x
12	120.13	42.0	2,002	x
13	120.42	41.9	2,127	
14	124.90	41.4	2,111	
15	104.04	42.2	1,620	x
16	98.21	41.2	1,367	x
17	94.99	42.9	2,005	
18	90.70	41.7	1,218	x
19	61.43	43.1	1,283	x
X	171,03	39.2	2,291	
Y	91,74	36.7	423	

Concerning heterologous associations, the Wald's asymptotic method was used to calculate 95% confidence intervals for the overlap of each pair of chromosomes. For those with an overlap of 0%, Wilsons’ score was used. Chromosomes with a confidence interval above or below the overall weighted mean of associations were considered statistically significant.

Analyses were performed by SAS v9.4 software, SAS Institute Inc., Cary, NC, EEU and the significance level was set to 0.05.

### Lymphocyte analysis

As somatic pairing does not occur in mice cells, we additionally analyzed the distribution of homologous chromosomes in lymphocytes, in order to obtain a basal level at which two homologous chromosomes are observed nearby due to non-pairing related causes. Accordingly, spleens were removed from two C57BL/6 J mice. After injecting 5 ml of RPMI into the spleens using a syringe and recovering the solution, lymphocytes were isolated by Ficoll-Paque gradient separation, fixed with 4% paraformaldehyde, and subjected to permeation treatment with 0.1 N hydrochloric acid, 0.5% triton, liquid nitrogen, and 0.005% pepsin (protocol adapted from Cremer et al. 2008). Next, FISH was again performed using the custom designed Chromoprobe Multiprobe® OctoChrome Murine System ™ kit (Cytocell Ltd, Cambridge, UK). In this case, only one coverslip was used; specifically, this coverslip permitted labeling the following chromosomes: 1, 3, 4, 6, 7, 8, 9, 10, 11, 12, 14, 16, 17, 18, and 19. The analysis was performed using an Olympus BX60 epifluorescence microscope equipped with filter sets for FITC, Texas Red, Aqua DEAC, and DAPI/Texas Red/FITC. The same criteria applied in the germ cell analysis were then used in order to classify the homologous chromosomes as paired or unpaired.

## Results

### Spermatogenetic cells

Application of the developed methodology allowed us to analyze the chromosome territories in a total of 147 spermatogonia-early preleptotene spermatocytes (premeiotic cells), 128 mid preleptotene-zygotene spermatocytes, 154 pachytene spermatocytes, and 321 round spermatids (Table 2). Considering all four cell fractions together, we analyzed a total of 5631 chromosome territories.

**Table 2:** FISH results for each chromosome in the four analyzed meiotic intervals

	Spermatogonia-early preleptotene (147)					Mid preleptotene-zygotene (128)					Pachytene (154)					Round spermatids (321)				
	2 signals		1 signal		Total	2 signals		1 signal		Total	2 signals		1 signal		Total	2 signals		1 signal		Total
Chr	*n*	%	*n*	%	*n*	*n*	%	*n*	%	*n*	*n*	%	*n*	%	*n*	*n*	%	*n*	%	*n*
1	19	30.16%	44	69.84%	63	14	24.56%	43	75.44%	57	0	0	52	100%	52	0	0	113	100%	113
2	13	23.64%	42	76.36%	55	5	12.82%	34	87.18%	39	0	0	52	100%	52	0	0	108	100%	108
3	14	22.58%	48	77.42%	62	11	22.00%	39	78.00%	50	0	0	59	100%	59	0	0	100	100%	100
4	19	29.69%	45	70.31%	64	12	20.34%	47	79.66%	59	0	0	55	100%	55	0	0	121	100%	121
5	20	35.71%	36	64.29%	56	4	9.76%	37	90.24%	41	0	0	51	100%	51	0	0	109	100%	109
6	14	28.57%	35	71.43%	49	7	14.58%	41	85.42%	48	0	0	50	100%	50	0	0	130	100%	130
7	20	30.77%	45	69.23%	65	11	22.45%	38	77.55%	49	0	0	60	100%	60	0	0	100	100%	100
8	12	25.53%	35	74.47%	47	8	18.18%	36	81.82%	44	0	0	48	100%	48	0	0	126	100%	126
9	14	31.11%	31	68.89%	45	8	16.33%	41	83.67%	49	0	0	58	100%	58	0	0	123	100%	123
10	14	28.00%	36	72.00%	50	11	20.75%	42	79.25%	53	0	0	62	100%	62	0	0	123	100%	123
11	5	14.29%	30	85.71%	35	4	7.84%	47	92.16%	51	0	0	57	100%	57	0	0	113	100%	113
12	11	26.83%	30	73.17%	41	4	7.84%	47	92.16%	51	0	0	59	100%	59	0	0	118	100%	118
13	9	21.95%	32	78.05%	41	12	23.53%	39	76.47%	51	0	0	43	100%	43	0	0	104	100%	104
14	16	25.40%	47	74.60%	63	7	11.86%	52	88.14%	59	0	0	54	100%	54	0	0	122	100%	122
15	12	28.57%	30	71.43%	42	4	12.12%	29	87.88%	33	0	0	40	100%	40	0	0	77	100%	77
16	11	20.75%	42	79.25%	53	10	21.28%	37	78.72%	47	0	0	59	100%	59	0	0	85	100%	85
17	9	25.71%	26	74.29%	35	4	11.11%	32	88.89%	36	0	0	40	100%	40	0	0	103	100%	103
18	11	37.93%	18	62.07%	29	10	20.00%	40	80.00%	50	0	0	56	100%	56	0	0	97	100%	97
19	2	7.69%	24	92.31%	26	1	2.22%	44	97.78%	45	0	0	49	100%	49	0	0	76	100%	76
X	10	28.57%	25	71.43%	35	4	8.33%	44	91.67%	48	0	0	42	100%	42	0	0	52	100%	52
Y	10		25		35	4		44		48	0	0	42		42	0	0	52		52
Mean		26.17%		73.83%			15.40%		84.60%			0%		100%			0%		100%	
Std Dev		6.78		6.78			6.40		6.40			0		0			0		0	

**Fig. 3:**
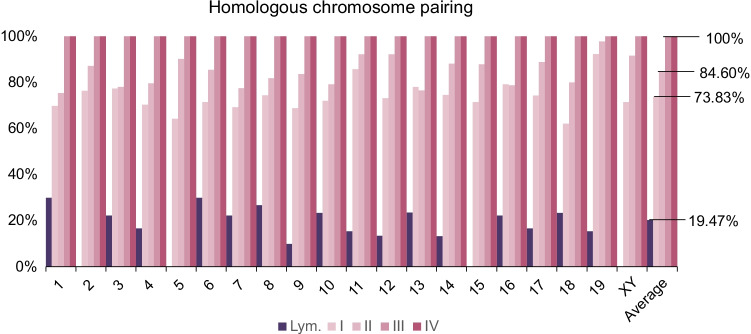
Percentage of homologous chromosome pairing observed in different stages of spermatogenesis: (I) spermatogonia-early preleptotene spermatocytes I, (II) spermatocytes I at mid preleptotene-zygotene stages, (III) spermatocytes I at pachytene stage, and (IV) round spermatids, as well as in lymphocytes (Lym.). In round spermatids (IV), the paired value corresponds to cells with one signal per chromosome

For each chromosome, the percentages of homologous chromosomes sharing the same territory (paired) or occupying different territories (unpaired) were represented for each cell stage analyzed in Fig. 3 (i.e., spermatogonia-early preleptotene spermatocytes, mid preleptotene-zygotene spermatocytes, pachytene spermatocytes, and round spermatids)**.** At the spermatogonium-preleptotene stage, a mean of 73.83% of homologous chromosomes was observed as a joint entity, this percentage increased up to 84.60% at the mid preleptotene-zygotene stage and reached 100% at the pachytene stage. As expected, round spermatids exhibited one signal per chromosome in 100% of the analyzed cells. Data showed differences in the pairing levels between chromosomes. For instance, chromosome 1 displayed one of the lowest pairing rates in the first two stages studied, specifically 69.84% and 75.44% of chromosomes were observed as paired homologous chromosomes in the spermatogonia-early preleptotene spermatocyte and mid preleptotene-zygotene spermatocyte stages, respectively. In contrast, chromosome 19 showed one of the highest pairing rates in these two stages; 92.31% and 97.78% respectively.

On average, the nuclear volume occupied by chromosomal territories classified as paired was higher than the nuclear volume occupied by unpaired signals, both in the spermatogonia-early preleptotene spermatocyte (1.79% for paired vs 1.11% for unpaired) and mid preleptotene-zygotene spermatocyte (2.37% vs 1.30%) stages (Table 3).

**Table 3:** Nuclear volume proportion (NVP) occupied by one signal composed by one chromosome (unpaired chromosome) or one signal composed by two chromosomes (paired chromosomes) in spermatogonia-early preleptotene and in mid preleptotene-zygotene germ cells. (*Chr*.) chromosomes, (*N*) number of observations

	Spermatogonia-early preleptotene				Mid preleptotene-zygotene			
	One signal composed by:				One signal composed by:			
	One chr		Two chr		One chr		Two chr	
Cr	*N*	NVP (%)	*N*	NVP (%)	*N*	NVP (%)	*N*	NVP (%)
1	38	1.98	44	1.85	28	1.94	43	2.44
2	26	1.09	42	2.14	10	1.24	34	3.59
3	28	1.21	48	2.07	22	1.22	39	3.66
4	38	1.67	45	1.96	24	1.22	47	3.00
5	40	0.78	36	2.23	8	0.94	37	2.47
6	28	1.11	35	1.84	14	1.76	41	2.76
7	40	0.91	45	2.04	22	1.76	38	2.74
8	24	0.97	35	1.87	16	0.78	36	1.97
9	28	0.53	31	1.21	16	0.86	41	1.91
10	28	1.21	36	1.82	22	1.70	42	2.91
11	10	1.08	30	1.62	8	1.22	47	1.87
12	22	1.23	30	2.63	8	1.58	47	3.02
13	18	1.68	32	2.14	24	0.65	39	2.44
14	32	1.28	47	1.83	14	1.08	52	2.29
15	24	0.64	30	1.18	8	0.99	29	1.36
16	22	1.16	42	1.48	20	1.59	37	2.07
17	18	0.56	26	1.31	8	1.00	32	1.53
18	22	0.78	18	1.21	20	1.18	40	1.68
19	4	0.38	24	0.84	2	1.46	44	1.38
X	10	1.62	25	1.72	4	1.05	44	2.84
Y	10	1.40	25	2.60	4	2.02	44	1.86
**Average**		**1.11**		**1.79**		**1.30**		**2.37**

To investigate possible explanations for the differences observed between chromosomes, Pearson’s correlation coefficient testing and/or *T*-tests were performed between the pairing rates and various intrinsic chromosomal parameters (Table 1). Results revealed that none of the chromosomal parameters analyzed was associated with the timing of homologous pairing, neither at the spermatogonia-early preleptotene spermatocyte stage (size: *r* =  − 0.36, *p* = 0.117; % GC: *r* = 0.21, *p* = 0.365; gene density: *r* =  − 0.12, *p* = 0.616) nor at the mid preleptotene-zygotene spermatocyte stage (size: *r* =  − 0.41, *p* = 0.070; % GC: *r* = 0.12, *p* = 0.616; gene density: *r* =  − 0.24, *p* = 0.318). *T*-test analysis revealed no significant differences between NOR-bearing-chromosomes and the remaining chromosomes either at the spermatogonia-early preleptotene spermatocyte stage (*p* = 0.135) or at the mid preleptotene-zygotene spermatocyte stage (*p* = 0.110).

The mean frequency of nonhomologous association was 41.10% in spermatogonia-early preleptotene spermatocyte stage. Data representing the frequencies at which nonhomologous chromosomes shared the same territory at this stage are detailed in Table 4. Ten non-homologous chromosome pairs out of 190 showed statistically significant increases with respect to the mean, while significant decreases were observed in 13 of them. Besides, data representing the degree of overlap between nonhomologous chromosomes are detailed in Suppl. Table 1. On average, chromosomes overlap 2.60% of its volume with other chromosomes. In the case of the ten nonhomologous chromosome pairs with significant increases associations, this percentage rises up to 4.1%. This percentage decreases to 0.95% in the case of nonhomologous chromosome pairs that interact less than the mean.

**Table 4:**
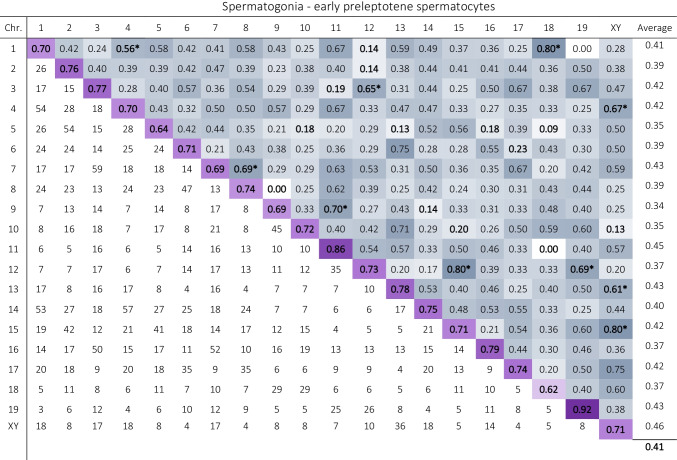
Data representing the frequencies and number of observations (*n*) of nonhomologous associations between all pairs of chromosomes at the spermatogonium-early preleptotene spermatocyte stage. At the top of the table (above the purple diagonal), the bluish tones indicate the frequency of overlap between all pairs of chromosomes, the higher the frequency of pairing, the more intense the blue coloration. Significant increases of associations are bolded and asterisked, whereas significant decreases are only bolded. The average pairing for each chromosome with all other nonhomologous chromosomes is indicated in the last column of the table. The number of observations made for each pair of chromosomes is indicated below the purple diagonal. Data in the mid-purple diagonal indicates the frequency of homologous pairing (the higher the frequency the more intense the purple coloration)

### Somatic cells

**Fig. 4:**
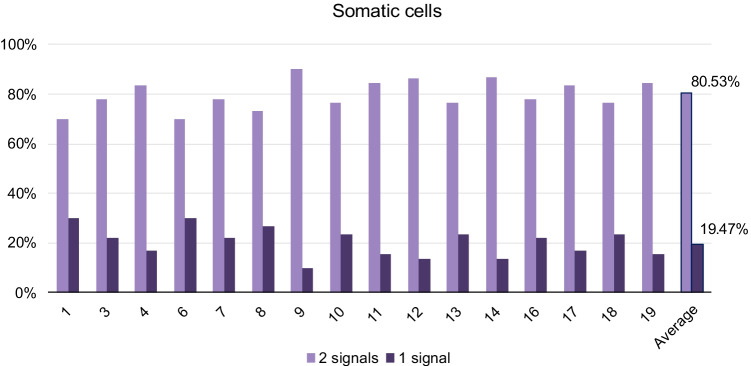
Percentage of homologous chromosomes occupying the same territory (one signal) or occupying different territories (two signals) for each chromosome in mice lymphocytes

One hundred forty-seven nuclei of mouse lymphocyte cells were analyzed, totalling 946 chromosome territories. A mean value of 19.47% of homologous chromosomes was observed as a joined entity (Fig. 3). Detailed data for each chromosome are graphically represented in Fig. 4.

## Discussion

Two opposing hypotheses explain when homologous paring begins in meiosis: the first proposes that homologous chromosomes approach one another during the leptotene stage as a consequence of DSB formation and the establishment of the bouquet structure. Conversely, the second theory proposes that homologous chromosomes initiate pairing in the early stages of meiosis, before DSB formation.

**Fig. 5:**
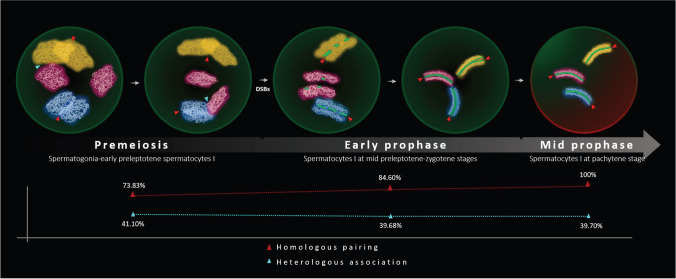
Schematic representation of the dynamics of homologous chromosome pairing and heterologous association from spermatogonia to pachytene spermatocytes of *Mus musculus.* There are represented three different homologous chromosomes in yellow, garnet, and blue colors. The homologous pairing is indicated by a red arrowhead and the heterologous association by a blue arrowhead. The nuclear background color indicates the presence of TRA98 (green) and H1T (red). The SYCP3 protein is represented in green dotted or continuous filaments

Our results indicate that, in the murine model, there is a high percentage of homologous chromosomes already sharing the same territory during the spermatogonium-preleptotene stage, prior to DSBs and bouquet formation Fig. 5. Therefore, our data suggest that pairing, defined as the approaching, juxtaposition, and overlapping of homologous chromosomes, is independent of both these processes. Our results agree with a previous study in which homologous paring for chromosome 3 in preleptotene spermatocytes were observed at a higher rate than heterologous interactions between chromosomes 3 and 7 (35% v.s 8% respectively; Boateng et al. 2013). Although the percentage of pairing in Boateng’s study is lower than the described by us for the same chromosomes (77.42% for homologous 3 chromosome pairing and 36% for heterologous interactions between 3 and 7) (Tables 2 and 4), we have to keep in mind that a different set of probes were used in both studies. The employment of a painting method that allows pairing-analysis along the entire chromosome, rather than a method based in the use of a single interstitial probe (Boateng et al. 2013), predicts an increase in the frequency of chromosomal-chromosome interactions. Boateng’s findings also revealed that premeiotic homologous contacts are lost at leptotene in the interstitial chromosome regions, but persist at telomeres, implying that telomeric interactions are stabilized and essential for future synapsis and recombination events. Unfortunately, our design, which relied on the employment of painting probes, prevented us from confirming these findings.

Our experimental design included various analyses whose results supported our main conclusion. First, we analyzed the homologous chromosome position in lymphocytes. Our results indicated that most homologous chromosomes occupy two different chromosomal territories in somatic cells, in agreement with several previous studies (Lorenz et al. 2003; Heride et al. 2010; Selvaraj et al. 2013; Rao et al. 2014; Joyce et al. 2016). Second, in this study, we determined that two homologous chromosomes were paired when they were overlapped and thus formed a unique entity. However, it is important to mention that the experimental design did not allow us to distinguish between those associations that resulted from pairing and those that resulted from the chromosomal topography of the nucleus. To clarify this point, we analyzed the frequency of nonhomologous association (interaction between non-homologous chromosomes). On average, none of the chromosomes reaches the association rate of the homologous one. Specifically, nonhomologous chromosomes showed an average association of about 41.1%, practically half the value observed in early meiotic stages Fig. 5. Third, we calculated the proportion of nuclear volume occupied by the paired (seemingly formed by two chromosomes) and the unpaired signals (each signal corresponding to one single chromosome) in premeiotic cells. Paired signals were, as a mean, 62% greater than those corresponding to unpaired signals, supporting the interpretation that paired signals corresponded to two homologous chromosomes. The fact that the ratio observed is not 2:1 can be interpreted into two different ways; chromosomes had a high percentage of overlap or paired chromosomes were more condensed.

Overall, these pieces of evidence suggest homologous chromosomes begin pairing before the onset of meiosis Fig. 5. The fact that there is a pairing of homologous chromosomes in pre-meiotic cells does not preclude the existence of a homologous pairing associated with the formation of DSBs. It is possible that chromosome pairing is carried out in two steps: a weak premeiotic pairing with a pre-juxtaposition function, and a robust late-meiotic pairing that is crucial for the upcoming events of synapsis and recombination.

Our data also bring information about the associations between nonhomologous chromosomes before the onset of meiosis. As previously indicated, we identified a mean of 41.10% non-homologous chromosome interactions, with certain pairs displaying significant increases or decreases, also exhibiting higher or lower percentage of overlapping. This result points out that in these particular cases, there might be some mechanisms increasing or reducing the likelihood of associations. Associations could be related to some intrinsic chromosome features such as size, %GC, gene density, or presence of NOR (Table 1). Nevertheless, the analysis of the chromosome pairs that showed differences in comparison to the mean ruled out this scenario (data not shown). Another mechanism could be related to the occurrence of interchromosomal contacts related to gene expression (Maass et al. 2019). Regarding this possibility, it has been described in the murine meiosis, a preferred functional association between some heterologous chromosomes using conformation capture sequencing (Hi-C); studies that include the analysis of spermatogonia (Vara et al., 2019).

The molecular mechanism that drives homologous chromosomes to approach each other prior to DSB formation is still unknown. However, several pieces of data have described different parameters that take part in this process (Barzel and Kupiec 2008; Ding et al. 2012; Boateng et al. 2013; Ishiguro et al. 2014). For instance, some studies in mice (Boateng et al. 2013; Ishiguro et al. 2014) have observed that the presence of Spo11 protein is involved in early pairing and synapsis. Additionally, Ishiguro et al. (2014) observed that disruption of RAD21L (a specific component of some meiotic cohesin complexes) causes a defect in early homolog pairing, bouquet stage arrest, and aberrant synapsis and postulated that early homolog recognition is achieved by the recognition of specific chromosome architecture rather than the homology of the DNA sequences. In other models, such as *Schizosaccharomyces pombe*, noncoding RNA transcripts accumulate at their respective gene loci to promote pairing of homologous loci in early prophase (Ding et al. 2012). In any case, further studies will be needed to complete the puzzle of the molecular mechanisms related to pre-DSB homologous pairing and to determine whether these are species-specific mechanisms or evolutionarily conserved processes.

Surprisingly, the high percentage of pairing observed in our results is contradicted by other FISH studies. We propose two different reasons to explain this situation: first, the methodologies used in these studies are based on the use of locus-specific probes (Scherthan et al. 1996; Scherthan and Schönborn, 2001). Therefore, they do not allow identify the entire territory of the chromosome and, thus, it is not possible to state with precision if two homologous chromosomes overlap. Secondly, only one or two chromosomes were analyzed in these studies and, therefore, the overall behavior of all chromosomes cannot be known (Scherthan et al. 1996; Scherthan and Schönborn, 2001; Ishiguro et al. 2014).

The main limitation of our experimental design is related to the impossibility of distinguishing between spermatogonia and the early-preleptotene stage. The initial spermatogonium-preleptotene stage includes types A and B spermatogonia, as well as spermatocytes I in the initial preleptotene stage. Nevertheless, it is important to mention that the expected ratio of type A spermatogonia, type B spermatogonia, and preleptotene spermatocytes in the mice testis is approximately 1: 1: 3 (Oakberg 1956; de Rooij 2001; Marchetti et al. 2018). Accordingly, it can be assumed that most nuclei analyzed in this interval correspond to early preleptotene spermatocytes, which are at the onset of the gradual approximation of homologous chromosomes in order to pair. In fact, the presence of spermatogonia in this interval, as well as the clear association of homologous chromosomes observed, opens the possibility that this association rather starts before the onset of meiosis. In any case, additional studies that split the populations of premeiotic cells into various pure fractions will be needed to clarify this point. Likewise, it has to be mentioned that the spermatocytes I at mid preleptotene-zygotene interval also involved different stages. If our experimental design had been able to distinguish among them, we would have probably observed a gradual increase of homologous pairing reaching up to 100% at zygotene. In fact, in the case of pachytene spermatocytes (when full synapsis occurs), our results indicate that 100% of homologous chromosomes are joined. Reinforcing this hypothesis, Scherthan et al. (1996) observe homologous chromosomes together in 100% zygotene and pachytene spermatocytes.

Our results point out that DSB-independent pairing does not occur between all chromosomes at the same time but instead, it is asynchronous Fig. 5. Asynchronous pairing processes have also been observed in other species (Rasmussen and Holm 1978; Guitart et al. 1985; Santos et al. 1993). Some studies have observed a relationship between the pairing timing and chromosome size. Specifically, smaller chromosomes initiate and complete their pairing earlier than the larger ones (Rasmussen and Holm 1978; Scherthan and Schönborn, 2001). In order to explain this association, it has been suggested that small chromosomes move more easily than the larger ones and, thus, complete the pairing process first. Alternatively, this early small-chromosome pairing could be a consequence of the association of the NOR-bearing chromosomes to form the nucleolus. However, our results do not indicate significant associations between size and pairing neither between NOR-bearing and non-NOR-bearing chromosomes and pairing. The fact that the mouse chromosome does not show large differences in size could be a reason for the lack of these associations.

In conclusion, our results confirm that there is a premeiotic chromosome pairing in murine germ cells. Future studies will be needed to determine the molecular mechanisms that regulate this process, as well as to elucidate any functions that this has in the spermatogenic process.

## Supplementary Information

Below is the link to the electronic supplementary material.

Supplementary file1 3D-FISH confocal image captures of each round of hybridization (a, b, c) of a spermatogonia-early preleptotene nucleus. Different combinations of three chromosomes (Chr.) displayed in FITC, Texas Red, or Aqua DEAC are observed in each round. For each hybridization round are shown: first, maximum intensity projections of confocal serial sections in RGB and scale grey; second, slice images segmentation by Fiji; and third, 3D composite reconstructions using Imaris 9.3 software in both RGB merge and split channels.   (MP4 22.6 MB)

Supplementary file2 Three-color 3D-FISH captures of mice germ cell nuclei. Different combinations of three chromosomes (Chr.) displayed in FITC (green), Texas Red (red), or Aqua DEAC (blue) are observed in three different premeiotic cell nuclei (a, b, c). Scale bar represents 5 µm. Images on the left show maximum intensity projections of confocal serial sections. Images on the right show the 3D composite reconstruction of the same nucleus using Imaris 9.3 software. Homologous chromosomes were classified as unpaired when two groups of pixels were observed as two separate entities or paired when all pixels of a specific signal formed a unique and continuous group of pixels. (MP4 17.0 MB)

Supplementary file3 Data representing the percentage of nonhomologous overlapping between all pairs of chromosomes at the spermatogonium-early preleptotene spermatocyte stage. There are two data for each pair of chromosomes. The percentage supper the empty diagonal refer to the territory of the chromosomes indicated in the first row. The percentages below the diagonal refer to the territory of the chromosomes indicated in the first column. The chromosomes pairs that showed significant increases of associations are bolded and asterisked, whereas significant decreases in associations are only bolded. The average of nonhomologous overlapping is 2.60. (DOCX 18.1 KB)

## Data Availability

“Not applicable.”

## Code availability

“Not applicable.”
