# Supplementary material for: Time to match; when do homologous chromosomes become closer?
Source: Chromosoma. 2022 Aug 12;131(4):193–205. doi: 10.1007/s00412-022-00777-0 (PMC9674740; doi:10.1007/s00412-022-00777-0)
Supplement: Supplementary file 3 — Supplementary file3 Data representing the percentage of nonhomologous overlapping between all pairs of chromosomes at the spermatogonium-early preleptotene spermatocyte stage. There are two data for each pair of chromosomes. The percentage supper the empty diagonal refer to the territory of the chromosomes indicated in the first row. The percentages below the diagonal refer to the territory of the chromosomes indicated in the first column. The chromosomes pairs that showed significant increases of associations are bolded and asterisked, whereas significant decreases in associations are only bolded. The average of nonhomologous overlapping is 2.60. (DOCX 18.1 KB) [file 412_2022_777_MOESM3_ESM.docx]

**Suppl. Table 1** Data representing the percentage of nonhomologous overlapping between all pairs of chromosomes at the spermatogonium-early preleptotene spermatocyte stage. There are two data for each pair of chromosomes. The percentages upper the empty diagonal refer to the territory of the chromosomes indicated in the first row. The percentages below the diagonal refer to the territory of the chromosomes indicated in the first column. The chromosomes pairs that showed significant increases of associations are bolded and asterisked, whereas significant decreases in associations are only bolded. The average of nonhomologous overlapping is 2.60.

|  |  |  |  |  |  |  |  |  |  |  |  |  |  |  |  |  |  |  |  |  |  |  |  |  |  |  |  |  |  |
| --- | --- | --- | --- | --- | --- | --- | --- | --- | --- | --- | --- | --- | --- | --- | --- | --- | --- | --- | --- | --- | --- | --- | --- | --- | --- | --- | --- | --- | --- |
| Chr. | 1 | 2 | 3 | 4 | 5 | 6 | 7 | 8 | 9 | 10 | 11 | 12 | | 13 | | 14 | | 15 | | 16 | | 17 | | 18 | | 19 | | XY | |
| 1 |  | 2.0 | 1.4 | **3.2*** | 3.4 | 1.2 | 0.3 | 2.4 | 6.0 | 0.9 | 2.3 | **4.4** | | 3.0 | | 7.8 | | 1.2 | | 5.2 | | 1.5 | | **0.1*** | | 0.0 | | 0.7 | |
| 2 | 3.6 |  | 2.1 | 4.5 | 1.8 | 0.5 | 2.0 | 1.2 | 0.0 | 0.6 | 0.9 | **0.0** | | 0.4 | | 0.9 | | 1.8 | | 0.8 | | 0.6 | | 0.4 | | 6.3 | | 0.4 | |
| 3 | 0.5 | 1.7 |  | 0.7 | 1.0 | 1.4 | 1.0 | 2.3 | 0.5 | 0.2 | **1.7** | **12.4*** | | 3.1 | | 2.0 | | 0.8 | | 2.5 | | 2.5 | | 2.7 | | 2.1 | | 2.1 | |
| 4 | **3.3*** | 3.5 | 1.0 |  | 3.8 | 0.5 | 12.9 | 6.0 | 0.7 | 0.7 | 3.4 | 0.1 | | 2.3 | | 2.3 | | 0.6 | | 2.4 | | 0.7 | | 0.7 | | 0.0 | | **3.7*** | |
| 5 | 3.3 | 1.6 | 0.8 | 4.0 |  | 2.5 | 1.8 | 1.8 | 3.0 | **0.7** | 0.0 | 1.7 | | **1.5** | | 2.1 | | 3.3 | | **1.5** | | 0.7 | | **0.3** | | 2.6 | | 2.4 | |
| 6 | 1.4 | 1.3 | 1.4 | 3.2 | 5.6 |  | 0.4 | 1.8 | 0.7 | 3.1 | 0.9 | 1.6 | | 4.3 | | 0.7 | | 0.9 | | 1.4 | | **1.0** | | 1.6 | | 0.4 | | 0.2 | |
| 7 | 0.4 | 2.4 | 1.4 | 9.9 | 1.7 | 1.1 |  | **9.2*** | 1.1 | 1.2 | 15.0 | 4.2 | | 1.1 | | 1.9 | | 2.4 | | 2.3 | | 0.7 | | 0.0 | | 0.5 | | 8.9 | |
| 8 | 4.7 | 1.5 | 2.7 | 3.3 | 3.4 | 1.6 | **7.1*** |  | **0.0** | 3.5 | 13.1 | 2.8 | | 1.2 | | 2.7 | | 0.1 | | 0.8 | | 1.2 | | 1.9 | | 1.1 | | 1.6 | |
| 9 | 2.7 | 0.1 | 0.9 | 1.3 | 0.7 | 2.9 | 2.0 | **0.0** |  | 0.9 | **0.6*** | 6.7 | | 2.0 | | **0.0** | | 0.2 | | 3.1 | | 0.5 | | 1.1 | | 0.0 | | 8.7 | |
| 10 | 0.7 | 8.0 | 0.2 | 0.1 | **0.3** | 5.3 | 0.1 | 3.6 | 0.6 |  | 0.4 | 3.8 | | 5.6 | | 0.9 | | **0.3** | | 0.4 | | 0.9 | | 2.9 | | 0.6 | | **0.0** | |
| 11 | 1.9 | 0.7 | **0.9** | 10.5 | 0.0 | 0.6 | 6.0 | 9.9 | **0.3*** | 1.2 |  | 2.7 | | 2.3 | | 0.0 | | 1.3 | | 3.6 | | 2.3 | | **0.0** | | 0.7 | | 1.3 | |
| 12 | **0.6** | **0.0** | **7.9*** | 0.1 | 4.6 | 0.9 | 3.6 | 3.4 | 2.9 | 5.8 | 2.3 |  | | 1.4 | | 2.2 | | **3.6*** | | 1.7 | | 1.3 | | 0.2 | | **2.3*** | | 7.0 | |
| 13 | 1.9 | 0.9 | 2.9 | 3.1 | **9.2** | 11.0 | 0.6 | 1.5 | 1.0 | 8.1 | 1.9 | 1.2 | |  | | 6.2 | | 0.7 | | 2.5 | | 0.5 | | 0.5 | | 2.5 | | **5.2*** | |
| 14 | 8.5 | 1.6 | 3.3 | 3.2 | 3.6 | 0.5 | 3.6 | 2.4 | **0.0** | 2.6 | 0.0 | 1.6 | | 8.1 | |  | | 3.2 | | 5.2 | | 4.5 | | 1.0 | | 5.5 | | 4.4 | |
| 15 | 4.4 | 3.0 | 1.6 | 0.5 | 2.7 | 1.6 | 1.0 | 0.1 | 0.2 | **0.2** | 1.4 | **2.6*** | | 0.4 | | 2.9 | |  | | 2.0 | | 5.7 | | 0.7 | | 2.2 | | **5.5*** | |
| 16 | 1.6 | 0.9 | 3.3 | 3.1 | **0.9** | 2.1 | 1.4 | 0.2 | 5.4 | 0.2 | 9.8 | 6.6 | | 8.0 | | 3.7 | | 0.2 | |  | | 7.0 | | 0.3 | | 4.0 | | 2.3 | |
| 17 | 2.2 | 1.3 | 6.4 | 1.5 | 2.1 | **1.2** | 1.7 | 3.3 | 0.6 | 2.8 | 5.1 | 8.4 | | 7.1 | | 10.5 | | 3.6 | | 4.5 | |  | | 0.0 | | 3.0 | | 0.1 | |
| 18 | **0.2*** | 0.4 | 5.4 | 2.8 | **0.1** | 9.1 | 0.0 | 3.9 | 1.1 | 8.1 | **0.0** | 0.1 | | 1.7 | | 3.1 | | 0.7 | | 0.4 | | 0.1 | |  | | 0.2 | | 0.1 | |
| 19 | 0.0 | 1.0 | 5.8 | 0.3 | 15.6 | 1.3 | 0.6 | 7.6 | 0.0 | 6.8 | 3.9 | **9.3*** | | 3.7 | | 17.5 | | 3.0 | | 3.8 | | 5.8 | | 0.2 | |  | | 10.6 | |
| XY | 0.2 | 0.1 | 2.5 | **4.2*** | 0.6 | 0.3 | 5.9 | 1.1 | 3.7 | **0.0** | 4.6 | 2.3 | | **5.6*** | | 2.8 | | **4.0*** | | 1.4 | | 0.0 | | 0.0 | | 3.7 | |  | |
|  |  |  |  |  |  |  |  |  |  |  |  |  |  | |  | |  | |  | |  | |  | |  | |  | |  |
